# Integrative Analysis of Metabolic Models – from Structure to Dynamics

**DOI:** 10.3389/fbioe.2014.00091

**Published:** 2015-01-26

**Authors:** Anja Hartmann, Falk Schreiber

**Affiliations:** ^1^Leibniz Institute of Plant Genetics and Crop Plant Research (IPK), Gatersleben, Germany; ^2^Monash University, Melbourne, VIC, Australia; ^3^Martin-Luther-University Halle-Wittenberg, Halle, Germany

**Keywords:** metabolic modeling, integrative analysis, kinetic analysis, flux balance analysis, petri net analysis, topological analysis

## Abstract

The characterization of biological systems with respect to their behavior and functionality based on versatile biochemical interactions is a major challenge. To understand these complex mechanisms at systems level modeling approaches are investigated. Different modeling formalisms allow metabolic models to be analyzed depending on the question to be solved, the biochemical knowledge and the availability of experimental data. Here, we describe a method for an integrative analysis of the structure and dynamics represented by qualitative and quantitative metabolic models. Using various formalisms, the metabolic model is analyzed from different perspectives. Determined structural and dynamic properties are visualized in the context of the metabolic model. Interaction techniques allow the exploration and visual analysis thereby leading to a broader understanding of the behavior and functionality of the underlying biological system. The System Biology Metabolic Model Framework (*SBM*^2^ – Framework) implements the developed method and, as an example, is applied for the integrative analysis of the crop plant potato.

## 1 Introduction

Metabolic models have been reconstructed for an increasing number of organisms to understand complex biochemical processes. At least 54 bacterial, 6 archaeal, and 16 eukaryotic reconstructions are available to-date while many others are under development (Xu et al., 2013). In addition, resources such as Path2Models (Büchel et al., 2013) provide draft models for a large number of organisms. Such metabolic models are composed of biochemical reactions and associated experimental parameters of the biological system under investigation. Different metabolic models can be reconstructed depending upon the completeness of knowledge about the detailed interaction mechanisms in a biological system. The metabolism is thereby roughly represented in large and mostly qualitative models and smaller, but more quantitative models (Steuer and Junker, 2008). Different model sizes and knowledge details allow the structural and dynamic properties to be analyzed using different modeling formalisms. For further details on modeling formalisms in Systems Biology the reader is referred to (Machado et al., 2011). Several modeling formalisms entail different analysis techniques facilitating the investigation of a metabolic model from different perspectives and thus, revealing complementary insights.

**Figure 1:**
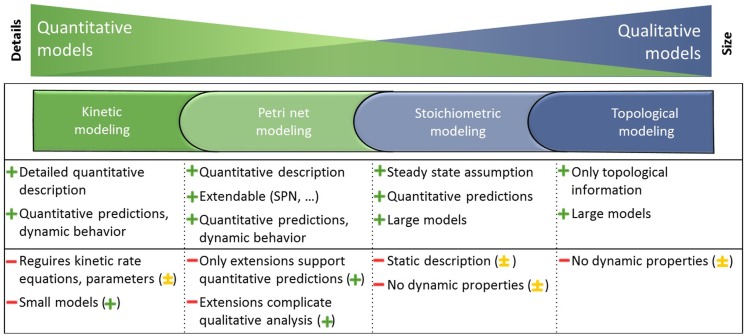
**Metabolism is represented in large and mostly qualitative models and smaller, but more quantitative models**. Different modeling formalisms that depend on the completeness of knowledge about the detailed interaction mechanisms are utilized to gain knowledge on the underlying biological system. Each modeling formalism is applied to different models as indicated by the corresponding colors and possesses strengths (+) and weaknesses (−). The integration of the independent modeling formalisms mitigates the weaknesses (−) and leads to the potential of the integrated analysis indicated in parentheses (major + or minor ± improvement, explanations are given in the Results and Discussion section). Adapted from Steuer and Junker (2008).

A couple of review papers evaluated modeling formalisms (Wiechert, 2002; Steuer and Junker, 2008; Hübner et al., 2011; Koch et al., 2011; Machado et al., 2011; Pfau et al., 2011; Dandekar et al., 2012) and revealed among others kinetic, Petri net, stoichiometric, and topological modeling methods as well-established. The strengths and weaknesses of each formalism are summarized in Figure 1.

Kinetic modeling using ordinary differential equations (ODEs) includes detailed quantitative descriptions on the biochemical processes and therefore requires often difficult to obtain kinetic rate equations and parameters. Due to this, kinetic modeling is generally limited to smaller models, but leads to quantitative predictions and reveals dynamic behavior of the underlying biological system (Resat et al., 2009). Petri net modeling is powerful due to several Petri net extensions for qualitative and quantitative analysis. The stochastic effects involved in quantitative predictions and system dynamics can be accounted for by using, for example, the stochastic Petri net (SPN) simulation. However, these extensions complicate the qualitative analysis (Baldan et al., 2010). Stoichiometric modeling using optimization-based analysis such as flux balance analysis (FBA) (Orth et al., 2010) allows for quantitative predictions due to the steady-state assumption. A static description of the biochemical processes is therefore sufficient when including stoichiometric, thermodynamic, and enzyme capacity constraints. Thus, stoichiometric modeling is applicable for large models, but is limited in revealing the dynamic behavior of the underlying biological system (Lewis et al., 2012). Topological modeling considers only the topological information of models (not limited in model size) and can identify structures and robustness against disturbances. Using, for example, centrality analysis (Koschützki and Schreiber, 2008) different importance concepts provide insights into key elements based on metabolite or reaction graphs (Steuer and Junker, 2008).

Some of the introduced metabolic modeling formalisms are already investigated in different approaches to analyze metabolic models at the system level and to overcome problems due to the lack of experimental data. Described methods either extent qualitative models with obtained analysis results to investigate a follow-up quantitative analysis, or models are reduced to assign less data for quantitative analysis. In most cases, such as Birch et al. (2014) and Chowdhury et al. (2014), the stoichiometric formalism FBA is used to obtain flux distributions, which are utilized to derive ODEs for kinetic analysis (Resat et al., 2009). Methods using the Petri net formalism for model reduction to integrate less data for kinetic analysis are described by Chen et al. (2011), Gilbert and Heiner (2006), and Koch and Heiner (2008). An advanced method is presented by Machado et al. (2010) whereby Petri net formalism is applied to integrate both of the aforementioned methods for model reduction and a follow-up kinetic analysis. Grafahrend-Belau et al. (2013) combined overview kinetic models (household models) with FBA toward a quasi-dynamic FBA. Heiner et al. (2012) and Nagasaki et al. (2010) propose a unifying Petri net framework comprised of a family of related Petri net types. In this approach qualitative, stochastic and continuous Petri net analyses are conducted by converting different Petri net types into each other.

Here, we introduce an integrated approach, which complements the presented approaches through a formalization leading to a standardized, transformable, and extensible abstraction of metabolism. This method allows the investigated metabolic models to be integrated, utilizing different well-established modeling formalisms and at the same time maintaining a standardized visualization. Moreover, the integration of analysis results with corresponding elements of the metabolic model leads to a combination of model structure and model dynamics. Several interaction techniques support the exploration and interpretation of the gained analysis results to provide a comprehensive understanding of the underlying biological system.

## 2 Materials and Methods

In general, metabolic models are networks consisting of different elements such as metabolites and reactions with relations between these elements and additional attributes. Thus, a suitable data structure for metabolic models is a graph. Dependent upon the modeling formalism, graphs with different structure and attributes are able to represent kinetic, Petri net, stoichiometric, or topological models. Each of these graphs contains nodes (metabolites and/or reactions), which are related to each other through edges.

**Figure 2:**
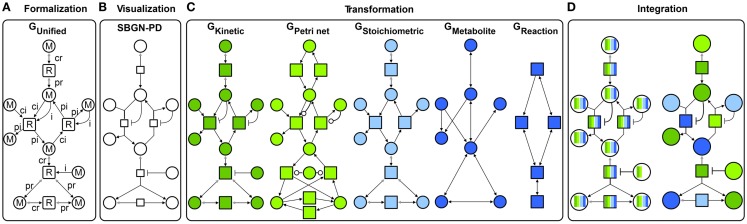
**Concept for an integrative analysis of metabolic models including**: **(A)** formalization (*G*_Unified_ with *M* metabolite and *R* reaction nodes, different edge types: *ci* consumption irreversible, *cr* consumption reversible, *pi* production irreversible, *pr* production reversible, and *i* inhibition), **(B)** visualization in *SBGN-PD*, **(C)** transformation in different *specific graphs*: *G*_Kinetic_ dark green, *G*_Petri net_ light green, *G*_Stoichiometric_ light blue, and two topological graphs *G*_Metabolite_ and *G*_Reaction_ dark blue, and **(D)** integration of different analysis results (colors represent results from different analysis performed using *specific graphs*).

Following the concept of generalization, different *specific graphs* representing qualitative and quantitative metabolic models (Figure 2C) are generalized into a *unified graph* (Figure 2A). This concept allows a standard graphical representation to be maintained (Figure 2B) and additionally, to transform the *unified graph* into *specific graphs* to apply different modeling formalisms. Some formalisms utilize a reduced structure and attribute set of the *unified graph* to perform analyses (this will be described in detail in the Transformation Section). Using our method, the analysis results from different formalisms are visualized in the context of the metabolic model through data assignment functions (Figure 2D). Thus, the underlying biological system is characterized from different perspectives providing complementary insights. Using interaction techniques, the subsequent visual analysis is conducted. Furthermore, analysis results can be integrated in other formalisms to constrain this analysis and thereby make them either feasible or more precise.

The following sections introduce the concept depicted in Figure 2 in detail.

### 2.1 Formalization

With the aim to formally represent qualitative and quantitative metabolic models a directed, attributed, bipartite graph (called the *unified graph*) is defined as follows.

**Definition 2.1** (unified graph). The *unified graph G*_Unified_ = (*M*, *R*, *E*, *A*) is a directed, attributed, bipartite graph consisting of two finite, non-empty sets *M* of metabolites and *R* of reactions, whereby both sets are disjoint *M* ∩ *R* = ∅. Other finite sets are directed edges *E* ⊆ (*M × R*) ∪ (*R × M*) and attributes *A* = {*type*, *stoichiometry*, *localization*, *label*, *concentration*, *capacity*, *rate*, *boundaries*, *objective function*}, which are assigned to nodes and edges using the following functions:

- *type: E* → {*ci*, *pi*, *cr*, *pr*, *i*} is a function, which assigns a type to each edge (*ci* consumption irreversible, *pi* production irreversible, *cr* consumption reversible, *pr* production reversible, or *i* inhibition). A directed edge from a metabolite to a reaction is of type *ci*, *cr*, or *i* [i.e., ∀*e* ∈ (*M × R*): *type*(*e*) = *ci* ∨ *type*(*e*) = *cr* ∨ *type*(*e*) = *i*] and a directed edge from a reaction to a metabolite is of type *pi* or *pr* [i.e., ∀*e* ∈ (*R × M*): *type*(*e*) = *pi* ∨ *type*(*e*) = *pr*]. To easily distinguish between reversible and irreversible edges, reversible edges are illustrated using a double-headed arrow, with the black arrow-head denoting the main direction from substrate (consumed metabolite) to product (produced metabolite) of a reaction.
- *stoichiometry*: *E*′ → ℝ_>0_ is a function, which assigns a positive real number greater than 0 to each edge of type *ci*, *cr*, *pi*, or *pr* out of the set *E*′ = {*e* ∈ *E*| ¬ (*type*(*e*) = *i*)}.
- *label*: *M* ∪ *R* → Σ* is a function, which assigns a word over the alphabet to each metabolite and each reaction.
- *localization*: *M* → Σ* is a function, which assigns a word over the alphabet to each metabolite.
- *capacity*: *M* → ℝ_≥0_ ∪ {∞} is a function, which assigns a positive real number or infinity {∞} to each metabolite.
- *concentration*: *M* → ℝ_≥0_ is a function, which assigns a positive real number to each metabolite. Additionally, the concentration of a metabolite has to be less than or equal to the capacity of the metabolite, ∀*m* ∈ *M: concentration(m)* ≤ *capacity*(*m*).
- *rate*: *R* →{{*h*, *j*}, *h*, *j*, {}} is a function, which assigns a kinetic rate equation *j* ∈ *J*, whereby *J* is a set of all kinetic rate equations or a positive real number (stochastic rate) *h* ∈ ℝ_≥0_ or the empty set to each reaction.
- *boundaries*: *R* → (*lower*, *upper*), with *lower*, *upper* ∈ ℝ_≥0_, and *lower* ≤ *upper* is a function, which assigns an ordered pair of positive real numbers to each reaction, whereby the lower bound has to be smaller than or equal to the upper bound.
- *objective function*: *R* → {0, 1}, with ∀*r*, *r*′ ∈ *R*: *objective function* (*r*) = 1 ∧ *objective function* (*r*′) = 1 ⇒ r = r′, is a function, which assigns 0 or 1 to each reaction, whereby only one reaction receives the value 1 (for optimization).

Furthermore, the following requirements must be fulfilled:

For all reactions *r* ∈ *R* applies: (1) there exists at least one incoming and one outgoing edge (whereby the incoming edge is not of type *i*) and (2) if one incoming or outgoing edge is reversible (irreversible) than all incoming and outgoing edges are reversible (irreversible). With this rule a reaction is either connected to reversible edges or irreversible edges but not a combination of them.

Between a metabolite *m* ∈ *M* and a reaction *r* ∈ *R* there are at most two edges *e*, *e*′ ∈ *E* of different types. If two edges *e* and *e*′ connect *m* with *r* the type of *e* is *ci* and the type of *e*′ is *i*. This case describes a substrate inhibition at high substrate concentrations, whereby a metabolite is substrate and inhibitor at the same time.

If one edge *e* connects *r* with *m* and another edge *e*′ connects *m* with *r* the type of *e* is *pi* and the type of *e*′ is *i*. In this case, a product inhibition is modeled with a metabolite as product and at the same time inhibitor of a reaction.

An explicit formulation of both cases for reversible reactions is not needed because the reaction mechanisms already provide implicit substrate- and product inhibition.

Moreover, the following sets are defined to simplify the transformation of the *unified graph* into *specific graphs* for analysis. The edge set *E* is composed of three subsets, *E* = *E_i_* ∪ *E_ir_* ∪ *E_r_*. The subset of inhibitory edges is *E_i_* = {*e* ∈ *E*|*type*(*e*) = *i*}, the subset of irreversible edges is *E_ir_* = {*e* ∈ *E|type*(*e*) = *ci* ∨ *type*(*e*) = *pi*} and the subset of reversible edges is *E_r_* = {*e* ∈ *E|type*(*e*) = *cr* ∨ *type*(*e*) = *pr*}. The set of metabolites *M* consists of a subset of metabolites *M_cp_*, which are either consumed or produced in reactions *M_cp_* = {*m* ∈ *M*|∃*r* ∈ *R*: (*m*, *r*) ∈ *E_r_* ∨ (*m*, *r*) ∈ *E_ir_*} ∪ {*m*′ ∈ *M*|∃*r* ∈ *R*:(*r*, *m*′) ∈ E_r_ ∨(r, m′) ∈ E_ir_}.

To assign analysis results to nodes and edges of the *unified graph*, data assignment functions that integrate calculated structural and dynamic data are used (this will be described in detail in the Transformation section).

**Figure 3:**
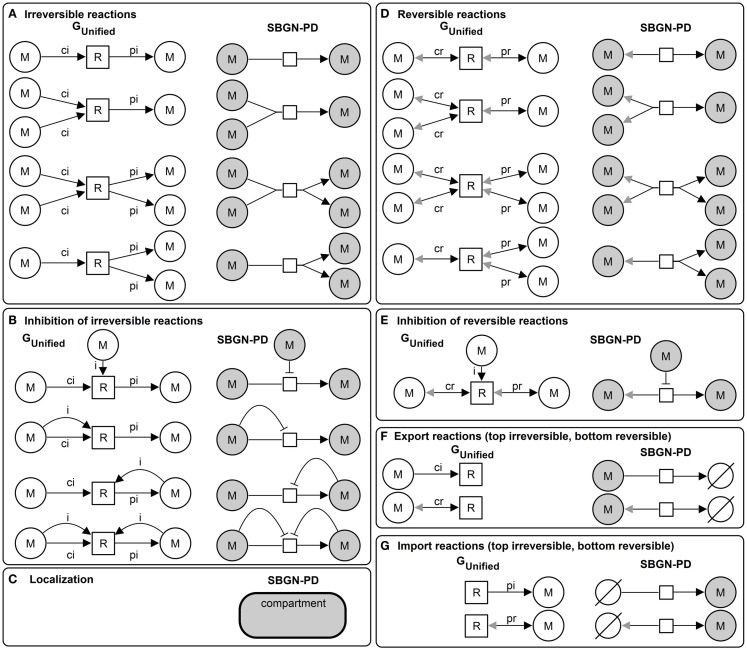
**Basic elements of the *unified graph* (left) and the corresponding *SBGN-PD* visualization (right)**: **(A)** irreversible reactions, **(B)** inhibition of irreversible reactions, **(C)** localization (compartment) of metabolites (samecolor), **(D)** reversible reactions, **(E)** inhibition of reversible reactions, **(F)** export reactions (top irreversible and bottom reversible), and **(G)** import reactions (top irreversible, bottom reversible).

Due to the definition of the *unified graph* with a rich attribute set qualitative and quantitative metabolic models can be represented and additionally visualized using standards. Figure 3 illustrates the basic elements of the *unified graph* and the corresponding visualization in *SBGN-PD*.

### 2.2 Visualization

In order to derive a standardized graphical representation of the *unified graph* the Systems Biology Graphical Notation (Le Novère et al., 2009) (*SBGN*) is utilized. *SBGN* has been developed to interpret biological models easily without the need for extensive descriptions using three sub-languages. *SBGN-PD* (Moodie et al., 2011) is the *Process Description* sub-language visualizing the temporal dependencies of biological interactions in detail and is thus suited for the metabolic models encoded in the *unified graph*.

The translation of the *unified graph* in a *SBGN-PD* visualization is based on the following schema. All elements of the metabolite set *m* ∈ *M* (reaction set *r* ∈ *R*) are visualized using *simple chemicals* ∈ *entity pool nodes* (*process* ∈ *process nodes*). All elements of the edge set *e* ∈ *E* are visualized using arcs of the set *connecting arcs* based on the assigned type. Edges of type *ci* are visualized using *consumption arc*, *pi* using *production arc*, *cr* using *production arc* in the opposite direction, *pr* using *production arc* and *i* using *inhibition arc*, respectively.

The edge attribute *stoichiometry* is visualized using *cardinality* and the metabolite attribute *localization* is visualized using *compartment*, which is a container for metabolites defined for this location. The localization of reactions is independent of a *compartment*, hence, a reaction could be located within, outside or on top of the border of a *compartment*. Import or export reactions in *SBGN-PD* are defined using the additional symbol *source and sink* ∈ *entity pool nodes*, see Figure 3.

Furthermore, interaction techniques allow the exploration and subsequent visual analysis leading to a broader understanding of the behavior and functionality of the underlying biological system (which will be described in detail in the Results and Discussion section).

### 2.3 Transformation

Overall, five transformations from the *unified graph* (*G*_Unified_) into the *specific graphs* (*G*_Kinetic_, *G*_Petri net_, *G*_Stoichiometric_, *G*_Metabolite_, *G*_Reaction_) have to be performed as a prerequisite to analyze a metabolic model using different modeling formalisms. The different models, modeling formalisms and the transformation from *G*_Unified_ into *G*_Stoichiometric_ are described in the following. The transformations from *G*_Unified_ into *G*_Kinetic_, *G*_Petri net_, and into both of the topological graphs *G*_Metabolite_, *G*_Reaction_ are defined in the Supplementary Material.

#### 2.3.1 Kinetic model

A kinetic metabolic model (ODE model) consists of a structural description of relations between metabolites and reactions and is extended with detailed kinetic data including rate equations, metabolite concentrations, and additional kinetic parameters. The kinetic model is represented by the *kinetic graph* (*G*_Kinetic_), which is transformed from the *unified graph (G*_Unified_*)*, see Figure 2C and for details Definition 1.1 in Supplementary Material. This transformation results in no structural differences, but in a reduced attribute set.

To analyze the kinetic metabolic model its *kinetic graph* is converted in ODEs, which are numerically solved (Resat et al., 2009). Changes in metabolite concentrations and reaction rates over a period of time are obtained as the results of the analysis.

#### 2.3.2 Petri net model

A Petri net metabolic model can be defined using different Petri net types. Here, we refer to extended qualitative place/transition Petri nets (*eP/T nets*) and extended quantitative stochastic Petri nets (*eSPNs*). The extension includes continuous tokens (to model metabolite concentrations), continuous arc weights (to model non-integer stoichiometry), continuous place capacities (to model limited resources), and inhibitor arcs (to model inhibition). An inhibition is modeled using an inhibitor arc from a place to a transition meaning that the transition can only fire if no token is on that place. The transition may only fire when the place is empty.

Both Petri net types share the same structure, but *eSPNs* are specialized by weights for the exponentially distributed random variable (firing time) assigned to transitions. For further details on Petri nets for modeling metabolic models the reader is referred to Baldan et al. (2010). The Petri net model is represented by the *Petri net graph* (*G*_Petri net_), which is transformed from the *unified graph* (*G*_Unified_), see Figure 2C and for details Definition 1.2 and Figure S1 in Supplementary Material. This transformation results in structural differences (reversible reactions are represented using a pair of irreversible reactions for both directions) and a reduced attribute set.

A Petri net metabolic model can be analyzed qualitatively or quantitatively. For the qualitative analysis, the *Petri net graph* is converted into a linear equation system, which can be solved to derive invariants describing main pathways (T-invariants) or metabolite conservation (P-invariants) of a metabolic model [more details in Murata (1989), Baldan et al. (2010), and Reisig (2013)]. Furthermore, all possible states are calculated using the reachability analysis and if the reachability graph cannot be constructed then the coverability graph is calculated instead (infinite state-space). The main purpose of the quantitative analysis (simulation) of a Petri net metabolic model is to include stochastic effects. The reactions can additionally be weighted with reaction rates to conduct a more constraint stochastic simulation revealing changes in metabolite concentrations over a number of simulation steps.

#### 2.3.3 Stoichiometric model

Compared to both of the aforementioned models a stoichiometric model consists of stoichiometric reactions without quantities, such as metabolite concentrations, or reaction rates. Due to the steady-state assumption, the regulatory effects resulting from enzymes or inhibitors are neglected; see Orth et al. (2010) for more details.

**Definition 2.2 (stoichiometric graph)**. The *unified graph G*_Unified_ is transformed in a directed, attributed, bipartite *stoichiometric graph G*_Stoichiometric_ = (*M_S_*, *R_S_*, *E_S_*, *A_S_*) with a metabolite set *M_S_* = *M_cp_*, which is a subset of the set *M* in *G*_Unified_. Metabolites with only inhibitory interactions to reactions are not considered. The reaction set in *G*_Stoichiometric_ *R_S_* = *R* equals the reaction set *R* set in *G_Unified_* and the edge set in *G*_Stoichiometric_ *E_S_* = *E_ir_* ∪ *E_r_* is a subset of the set *E* in *G_Unified_*. Edges of type *i* are excluded. The attribute set in *G*_Stoichiometric_ *A_S_* ⊆ *A* is a subset of the set *A* in *G*_Unified_ with *A_S_* = {*type*, *stoichiometry*, *localization*, *label*, *boundaries*, *objective function*}.

Figure 2C and for details Figure S4 in Supplementary Material depict the transformation of inhibited reactions from *G*_Unified_ into *G*_Stoichiometric_ and thereby detailing the difference between both graphs. This transformation results in structural differences (no inhibitions) and a reduced attribute set. Thereby, all regulatory information and quantitative data are lost.

Using the *stoichiometric graph*, a metabolic model can be validated utilizing the *Dead-End* analysis or *Gap-Finding* analysis revealing blocked reactions or dead-end metabolites. To examine the flow of metabolites through a metabolic model the *stoichiometric graph* is converted into a system of mass balance equations at steady-state, which are solved by minimizing or maximizing an objective function. This optimization can be conducted using a linear optimization instead of a non-linear optimization to handle the problem of alternate optimal solutions. Applicable optimization-based methods are *FBA*, flux variability analysis (*FVA*), robustness analysis (*RA*), and knockout-analyses (*KA*) resulting in a flux distribution, minimal and maximal fluxes, sensitivity curves, and sensitivity values, respectively. For a detailed description of optimization-based methods the reader is referred to (Lewis et al., 2012).

#### 2.3.4 Topological models

Metabolic models are analyzed according to topological properties in order to understand the importance of key elements, structure, and robustness against disturbances. Since the *metabolite graph* (nodes represent metabolites, edges reactions) and *reaction graph* (nodes represent reactions, edges metabolites) are predominantly used for topological analysis (Steuer and Junker, 2008) the *unified graph G*_Unified_ is transformed into both, see Figure 2C (For details see Definition 1.3 and Figure S2 in Supplementary Material for *metabolite graph* and Definition 1.4 and Figure S3 in Supplementary Material for *reaction graph*). This transformation results in structural differences (unipartite graphs) and a reduced attribute set. Thereby, all regulatory information and quantitative data are lost.

Topological analysis of the metabolic model based on its *metabolite graph* or *reaction graph* is conducted using the corresponding adjacency matrix. A shortest path analysis results in paths (subgraphs which could be the graph itself). Furthermore, centrality analysis with different centrality measures leads to a ranking of graph elements according to different importance concepts. For further details on different centrality measures the reader is referred to Koschützki and Schreiber (2008).

### 2.4 Integration

To integrate structural and dynamic analysis results in the *unified graph*, which have been computed using *specific graphs*, data assignment functions are applied. To focus on several analysis methods, we chose typical examples from a number of analysis methods comprised in the different modeling formalisms. Using these analysis methods, two sets of data types are generated: vectors of numeric values and graph elements, which are assigned to different graph elements of the *unified graph*, see Table 1.

**Table 1:** **Summary of typical examples of analysis methods and corresponding results produced with different modeling formalisms grouped in data types, which will be assigned to different graph elements [metabolite nodes (*M*), reaction nodes (*R*), and edges (*E*)] of the *unified graph***.

Modeling formalisms	Typical examples of analysis methods		Analysis results	Data types	*G*_Unified_		
					*M*	*R*	*E*
Kinetic modeling	Kinetic analysis		Metabolite concentrations, reaction rates over time	Vector of time dependent numeric values	x	x	
Petri net modeling	Invariant analysis		P- and T-invariants	Vector of numeric values	x^a^	x^a^	
	Reachability analysis		Reachability graph/coverability graph	Graph	x^a^	x^a^	
	Stochastic analysis		Metabolite concentrations, reaction rates over steps	Vector of step dependent numeric values	x^a^	x^a^	
Stoichiometric modeling	Stoichiometric analysis		Dead-ends	Nodes	x		
	Optimization-based analysis	Gap-finding	Gaps	Nodes	x		
		FBA	Flux distribution	Vector of numeric values			x
		RA	Sensitivity curve	Vector of flux dependent numeric values		x	
		KA	Sensitivity value	Vector of numeric values		x	
		FVA	Min/max flux values of reactions	Vector of numeric value pairs		x	
Topological modeling	Centrality analysis		Centrality values	Vector of numeric values	x	x	
	Shortest path		Shortest path	Graph	x^b^	x^b^	x^b^

*^a^Analysis results from forward and backward reactions of the Petri net are integrated into the corresponding reversible reactions in the unified graph*.

*^b^Analysis results from edges of the metabolite graph or reaction graph correspond to several edges and nodes in the unified graph*.

Numeric values of the vector (*nv* ∈ *NV*) are assigned to elements of the *unified graph* (*M* metabolite, *R* reaction, and *E* edge) using the assignment function *zn*: *M*, *R*, *E* → *NV*, whereby the vector could comprise numeric values (e.g., sensitivity values), pairs of numeric values (e.g., min and max fluxes), and a set of time, step, and flux value dependent numeric values (e.g., metabolite concentrations over time, steps and sensitivity curves, respectively).

Another type of analysis results data are the elements of graphs, which are assigned to the *unified graph* using the assignment function *zg*: *M*, *R*, *E* → *M_x_*, *R_x_*, *E_x_*, whereby *x* can be replaced with *P* Petri net, *S* stoichiometric, *K* kinetic, *M* metabolite, or *R* reaction to define the *specific graphs*. As an example, *Gap-Finding* analysis results in a set of metabolites of the *stoichiometric graph*, which must be assigned to metabolites in the *unified graph* using *zg*: *M* → *M_S_*.

These assignment functions provide the basis for the visualization of the analysis results in the context of the metabolic model. Furthermore, interaction techniques such as *brushing* & *linking* and *animation* support the exploration, for example, of different Petri net invariants in the context of the metabolic model [for more details concerning interaction techniques see Von Landesberger et al. (2011)]. An integrated visualization by means of an application using the developed method is represented in the Results and Discussion section.

## 3 Results and Discussion

In conclusion, the developed method allows previously separated well-established modeling formalisms to be combined into one application using one workflow, supported by interaction techniques and integrated visualizations in the context of the metabolic model. The method mitigates the weaknesses (−) of independent modeling formalisms as explained in the Introduction section and leads to major (+) or minor (±) improvements of an integrated analysis as already depicted in Figure 1.

In detail, using the integrated approach it is not required to define detailed kinetics to derive quantitative predictions and reveal dynamic behavior of the underlying biological system. Instead, using some parameters the Petri net simulation or stoichiometric modeling method FBA could be performed to approximate kinetic simulations. Thus, larger models are applicable in the integrated approach leading to analysis results, which could be again integrated to analyze the model further. Additionally, qualitative analysis can be conducted for extended Petri nets using another integrated formalism such as Dead-End analysis or centrality analysis. Quantitative predictions can be revealed for a qualitative model with a static description using stoichiometric analysis.

Hence, different modeling formalisms complement each other even through, overlaps between the introduced metabolic modeling formalisms exist. For example, the stoichiometric matrix used in the stoichiometric modeling formalism to derive mass balance equations corresponds to the incidence matrix of the Petri net formalism used to derive an equation system solved for, e.g., invariant analysis. In the case of structural analysis, both the stoichiometric and the Petri net formalism could be utilized to reveal, for example, *Dead-End* metabolites. Additionally, Petri net T-invariants correspond to flux modes, which could be directly calculated using the stoichiometric analysis method elementary flux modes (not presented here).

The described method is implemented as an Add-on for the *VANTED* system (Rohn et al., 2012), called the System Biology Metabolic Model Framework (*SBM*^2^ – Framework). It utilizes and extends *VANTED*s functionality for the interpretation of experimental data and for analyzing metabolic models with different modeling formalisms.

In order to characterize the metabolic functionality and behavior of the crop plant potato (*Solanum tuberosum*) an integrative analysis is performed using the described method. Due to its main component, starch in the potato tuber, potato is of great importance as food and in industry, for example, for the production of fuel. Therefore, a major aim of plant breeding is to improve the distribution of biomass within the plant in favor of harvestable plant parts. Based on the homogeneous tissue of the potato tuber the main flux of metabolites is from sucrose to starch (Geigenberger et al., 2004). The investigation of sucrose degradation can be conducted. Almost all genes of this pathway are already known and thus provide the basis for the reconstruction of a metabolic model of the potato tuber.

**Figure 4:**
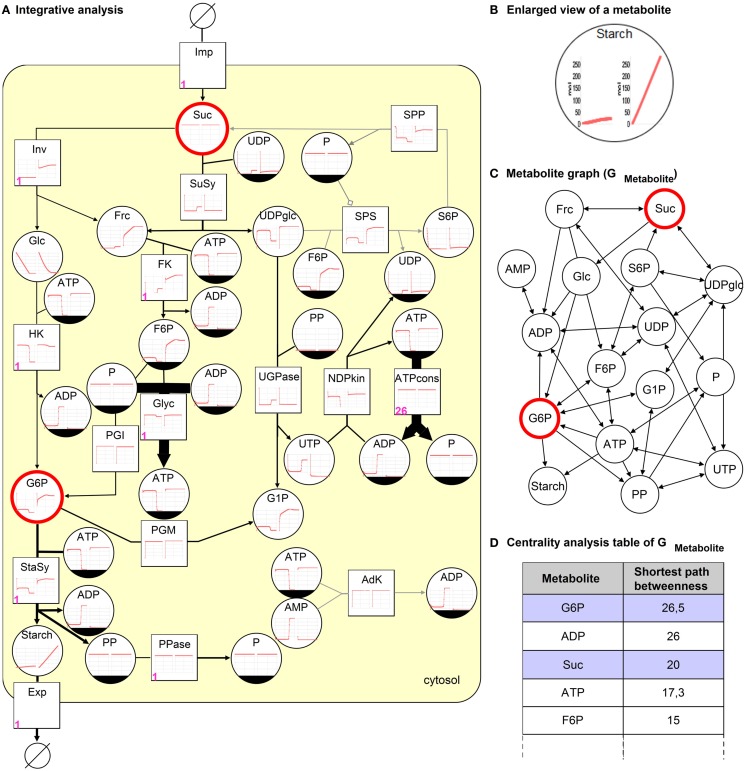
**Integrative analysis of the sucrose breakdown in the potato tuber**. **(A)** The kinetic analysis results in time-course diagrams of metabolites and reactions (left wild type, right overexpression of *Inv*), **(B)** enlarged view of both diagrams for metabolite starch. Petri net invariant analysis results in T-invariants, one is represented using numbers (firing counter, left lower corner in pink) assigned to reactions. The steady-state flux distribution resulting from FBA optimized for maximization of starch biosynthesis is depicted as edge thickness (gray edge indicates 0 flux). **(C)** The topological analysis (shortest path betweenness centrality analysis) of the *metabolite graph* results in a **(D)** ranked table. Two metabolites are selected in the table (blue), which correspond to the highlighted (red) nodes in **(A,C)**.

Using a kinetic model representing the sucrose breakdown in the developing potato tuber (Junker, 2004) the integrative analysis is performed and analysis results are shown in Figure 4A. The model comprises of 15 reactions and 17 metabolites located in the cytosol. Sucrose (*Suc*) is converted into hexose phosphates (e.g., glucose-6 phosphate, *G*6*P*) utilized in glycolysis (*Glyc*) and as precursors for starch synthase (*StaSy*). The pathways *Glyc*, starch biosynthesis, and energy consumption (*ATP*_cons_) are modeled as summarized reactions. This is a necessary simplification to avoid unknown transport processes into additional compartments. To describe the environment the model is extended through sucrose import (*Imp*) and starch export reactions (*Exp*).

The kinetic analysis results in time-course diagrams converging toward a steady-state producing starch, which can be increased by an overexpression of the enzyme invertase (*Inv*) as described in Junker (2004). The consequence of the overexpression can be compared and visually analyzed to investigate both situations side by side in the model, see Figures 4A,B.

To perform a stochastic simulation the steady-state reaction rates generated by the kinetic analysis are used to weight the reactions of the eSPN. The stochastic simulation results in increasing and decreasing metabolite concentrations, which oscillate with different amplitudes (data not shown). The results indicate the production of starch and the utilization of reactions with different probabilities.

Additionally, the invariant analysis reveals beside 3 P-invariants (reflecting substance conservation) 19 T-invariants, which can be grouped in trivial and non-trivial T-invariants. Each of the seven trivial T-invariants corresponds to a reversible reaction. The non-trivial T-invariants can be differentiated in a group of nine representing the cleavage of sucrose by invertase and another group of three where the sucrose is cleaved by sucrose synthase. These T-invariants reflect the main processes that are pathways taking place in the metabolic model in reality (Koch et al., 2005). One of the T-invariants is illustrated in Figure 4A by adding numbers (firing counter) to the corresponding reactions. Sucrose is initially cleaved by invertase, leading to the production of hexose phosphates, which are metabolized in *Glyc* and starch biosynthesis.

The stoichiometric analysis (irrespective regulatory processes), using only three steady-state reaction rates (*Inv* = 0.16 μM/FW/s, *SuSy* = 4.89 μM/FW/s, *ATPcons* = 100 μM/FW/s) to constrain the fluxes for these reactions, results in a flux distribution, which is comparable to the kinetic analysis results. In Figure 4A, the edge thickness corresponds to flux values. The flux through the starch biosynthesis reaction with 6.42 μM/FW/s is equal to the one of the kinetic analysis. Additionally, the reaction *AdK* is not utilized as can be seen in results of the kinetic and Petri net analysis.

Using the *metabolite graph*, see Figure 4C, the structure of the potato model is investigated. To identify important metabolites that occur on the shortest paths between two nodes in a ranked way the *shortest path betweenness (SPB)* centrality analysis is conducted. As a result, the table in Figure 4D illustrates *Suc* and *G*6*P*, which are selected to be highlighted in Figures 4A,C. Both metabolites are very important in the model, indicating that without these metabolites the reactions of starch biosynthesis and *Glyc* could not be processed.

In summary, using the integrative analysis allows different modeling formalisms to be investigated in one workflow. An integrated and interactive visualization of the analysis results leads to an advantage over the use of each modeling formalism independently. This helps to compare analysis results from different formalisms within one metabolic model and allows for the investigation of analysis results from one formalism in another, as mentioned in the use case.

## 4 Conclusion

We described a method, which is able to bring together different metabolic modeling formalisms. The integration is realized by a *unified graph*, enabling graph transformations, and a visualization in a standardized and formalized way. The *unified graph* supports user interaction and thereby allows different analysis results to be explored in the context of the metabolic model. The application reveals structural and dynamic properties of the crop plant potato utilizing the integrative analysis. The method has been implemented as an extension of the *VANTED* system and could also be applied to other model types, but we have focused here on metabolic models as an application area.

Combining different modeling formalisms opens many possibilities for future research. Additional analysis algorithms can be added to study metabolic models in more detail. We plan to extend the method for different types of models such as gene regulatory models to investigate further cellular processes. This extension requires the adaptation of the *unified graph*, adding of appropriate modeling formalisms, and corresponding transformations. Furthermore, the visualization has to be adapted to represent different types of models in *SBGN* using, for example, the sub-language *SBGN-AF* for gene regulatory models.

## Author Contributions

Anja Hartmann developed the theoretical framework, the use case, and implemented the *SBM*^2^ – Framework software. Falk Schreiber supervised the project and gave conceptual advice. Both authors wrote the manuscript.

## Conflict of Interest Statement

The authors declare that the research was conducted in the absence of any commercial or financial relationships that could be construed as a potential conflict of interest.

## Supplementary Material

The Supplementary Material for this article can be found online at http://www.frontiersin.org/Journal/10.3389/fbioe.2014.00091/abstract

## References

[B1] Baldan P. Cocco N. Marin A. Simeoni M. (2010). Petri nets for modelling metabolic pathways: a survey. Nat. Comput. 9, 955–989 10.1007/s11047-010-9180-6

[B2] Birch E. W. Udell M. Covert M. W. (2014). Incorporation of flexible objectives and time-linked simulation with flux balance analysis. J. Theor. Biol. 345, 12–21. 10.1016/j.jtbi.2013.12.009 24361328 PMC3933926

[B3] Büchel F. Rodriguez N. Swainston N. Wrzodek C. Czauderna T. Keller R. (2013). Path2models: large-scale generation of computational models from biochemical pathway maps. BMC Syst. Biol. 7:116. 10.1186/1752-0509-7-116 24180668 PMC4228421

[B4] Chen M. Hariharaputran S. Hofestädt R. Kormeier B. Spangardt S. (2011). Petri net models for the semi-automatic construction of large scale biological networks. Nat. Comput. 10, 1077–1097 10.1007/s11047-009-9151-y

[B5] Chowdhury A. Zomorrodi A. R. Maranas C. D. (2014). k-OptForce: integrating kinetics with flux balance analysis for strain design. PLoS Comput. Biol. 10:e1003487. 10.1371/journal.pcbi.1003487 24586136 PMC3930495

[B6] Dandekar T. Fieselmann A. Majeed S. Ahmed Z. (2012). Software applications toward quantitative metabolic flux analysis and modeling. Brief. Bioinformatics 15, 91–107. 10.1093/bib/bbs065 23142828

[B7] Geigenberger P. Stitt M. Fernie A. R. (2004). Metabolic control analysis and regulation of the conversion of sucrose to starch in growing potato tubers. Plant Cell Environ. 27, 655–673 10.1111/j.1365-3040.2004.01183.x

[B8] Gilbert D. Heiner M. (2006). “From Petri nets to differential equations – an integrative approach for biochemical network analysis,” in ICATPN, Volume 4024 of Lecture Notes in Computer Science, eds Donatelli S. Thiagarajan P. S. (Berlin: Springer), 181–200.

[B9] Grafahrend-Belau E. Junker A. Eschenröder A. Müller J. Schreiber F. Junker B. H. (2013). Multiscale metabolic modeling: dynamic flux balance analysis on a whole-plant scale. Plant Physiol. 163, 637–647. 10.1104/pp.113.224006 23926077 PMC3793045

[B10] Heiner M. Herajy M. Liu F. Rohr C. Schwarick M. (2012). “Snoopy – a unifying Petri net tool,” in Application and Theory of Petri Nets, Volume 7347 of Lecture Notes in Computer Science, eds Haddad S. Pomello L. (Berlin: Springer), 398–407.

[B11] Hübner K. Sahle S. Kummer U. (2011). Applications and trends in systems biology in biochemistry. FEBS J. 278, 2767–2857. 10.1111/j.1742-4658.2011.08217.x 21707921

[B12] Junker B. H. (2004). Sucrose Breakdown in the Potato Tuber. Dissertation, Faculty of Science, Potsdam: University of Potsdam.

[B13] Koch I. Heiner M. (2008). “Petri nets,” in Analysis of Biological Networks, eds Junker B. H. Schreiber F. (Wiley), 139–180.

[B14] Koch I. Junker B. H. Heiner M. (2005). Application of Petri net theory for modelling and validation of the sucrose breakdown pathway in the potato tuber. Bioinformatics 21, 1219–1226. 10.1093/bioinformatics/bti145 15546934

[B15] Koch I. Reisig W. Schreiber F. (2011). Modeling in Systems Biology: The Petri net Approach. New York, NY: Springer, 16.

[B16] Koschützki D. Schreiber F. (2008). Centrality analysis methods for biological networks and their application to gene regulatory networks. Gene Regul. Syst. Bio. 2, 193–201. 19787083 10.4137/grsb.s702 PMC2733090

[B17] Le Novère N. Hucka M. Mi H. Moodie S. Schreiber F. Sorokin A. (2009). The systems biology graphical notation. Nat. Biotechnol. 27, 735–741 10.1038/nbt0909-864d 19668183

[B18] Lewis N. E. Nagarajan H. Palsson B. O. (2012). Constraining the metabolic genotype-phenotype relationship using a phylogeny of in silico methods. Nat. Rev. Microbiol. 77, 541–580. 10.1038/nrmicro2737 22367118 PMC3536058

[B19] Machado D. Costa R. Rocha M. Ferreira E. C. Tidor B. Rocha I. (2011). Modeling formalisms in systems biology. AMB Express 1, 45–58 10.1186/2191-0855-1-45 22141422 PMC3285092

[B20] Machado D. Costa R. S. Rocha M. Rocha I. Tidor B. Ferreira E. C. (2010). “Model transformation of metabolic networks using a Petri net based framework,” in ACSD/Petri Nets Workshops, Volume 827 of CEUR Workshop Proceedings, eds Donatelli S. Kleijn J. Machado R. J. Fernandes J. M. (Braga: CEUR-WS.org), 103–117.

[B21] Moodie S. Novère N. L. Demir E. Mi H. Schreiber F. (2011). Systems biology graphical notation: process description language level 1. Nat. Proc. 10.1038/npre.2011.3721.4 26528561

[B22] Murata T. (1989). Petri nets: properties, analysis and applications. Proc. IEEE 10, 291–291.

[B23] Nagasaki M. Saito A. Jeong E. Li C. Kojima K. Ikeda E. (2010). Cell illustrator 4.0: a computational platform for systems biology. In silico Biol. 10, 5–26. 22430219 10.3233/ISB-2010-0415

[B24] Orth J. D. Thiele I. Palsson B. O. (2010). What is flux balance analysis? Nat. Biotechnol. 28, 245–248 10.1038/nbt.1614 20212490 PMC3108565

[B25] Pfau T. Christian N. Ebenhöh O. (2011). Systems approaches to modelling pathways and networks. Brief. Funct. Genomics 10, 266–279. 10.1093/bfgp/elr022 21903724

[B26] Reisig W. (2013). Understanding Petri Nets – Modeling Techniques, Analysis Methods, Case Studies. Berlin: Springer.

[B27] Resat H. Petzold L. Pettigrew M. F. (2009). “Kinetic modeling of biological systems,” in Computational Systems Biology., Volume 541 of Methods in Molecular Biology, eds Ireton R. Montgomery K. Bumgarner R. Samudrala R. McDermott J. (New York: Humana Press), 311–335. 10.1007/978-1-59745-243-4_14 PMC2877599 19381542

[B28] Rohn H. Junker A. Hartmann A. Grafahrend-Belau E. Treutler H. Klapperstück M. (2012). Vanted v2: a framework for systems biology applications. BMC Syst. Biol. 6:139. 10.1186/1752-0509-6-139 23140568 PMC3610154

[B29] Steuer R. Junker B. H. (2008). “Computational models of metabolism: stability and regulation in metabolic networks,” in Advances in Chemical Physics, Vol. 142, ed. Rice S. A. (Hoboken: John Wiley and Sons, Inc), 105–251 10.1002/9780470475935.ch3

[B30] Von Landesberger T. Kuijper A. Schreck T. Kohlhammer J. van Wijk J. Fekete J.-D. (2011). Visual analysis of large graphs: state-of-the-art and future research challenges. Comput. Graph. Forum 30, 1719–1749 10.1111/j.1467-8659.2011.01898.x

[B31] Wiechert W. (2002). Modeling and simulation: tools for metabolic engineering. J. Biotechnol. 94, 37–63 10.1016/S0168-1656(01)00418-7 11792451

[B32] Xu C. Liu L. Zhang Z. Jin D. Qiu J. Chen M. (2013). Genome-scale metabolic model in guiding metabolic engineering of microbial improvement. Appl. Microbiol. Biotechnol. 97, 519–539. 10.1007/s00253-012-4543-9 23188456

