# Supplementary material for: Integrative Analysis of Metabolic Models – from Structure to Dynamics
Source: Front Bioeng Biotechnol. 2015 Jan 26;2:91. doi: 10.3389/fbioe.2014.00091 (PMC4306315; doi:10.3389/fbioe.2014.00091)
Supplement: Supplementary file 1 [file Presentation_1.PDF]

Frontiers in Bioengineering

Methods Article
4 November 2014

Supplementary Material: Integrative Analysis of Metabolic Models - from Structure to Dynamics

Anja Hartmann [1,*] and Falk Schreiber [2,3]

[1] *Leibniz Institute of Plant Genetics and Crop Plant Research (IPK), Gatersleben, Germany*
[2] *Monash University, Melbourne, Victoria, Australia*
[3] *Martin-Luther-University Halle-Wittenberg, Halle, Germany*

Correspondence*:
Anja Hartmann
Leibniz Institute of Plant Genetics and Crop Plant Research (IPK), Corrensstr. 3, OT Gatersleben, Stadt Seeland, 06466, Germany, hartmann@ipk-gatersleben.de

Current challenges in modeling cellular metabolism

1 SUPPLEMENTARY DEFINITIONS

Transformations from the *unified graph* ($G_{Unified}$) into the *specific graphs* ($G_{Kinetic}$, $G_{Petri\,net}$, $G_{Stoichiometric}$, $G_{Metabolite}$, $G_{Reaction}$) have to be performed as prerequisite to analyze a metabolic model using different modeling formalisms. The different models, modeling formalisms and the transformation from $G_{Unified}$ into $G_{Stoichiometric}$ are described in the main article and illustrated in **Supplementary Figure 4**. Here, the transformations from $G_{Unified}$ into $G_{Kinetic}$, $G_{Petri\,net}$, and into the both topological graphs $G_{Metabolite}$, $G_{Reaction}$ are defined.

SUPPLEMENTARY DEFINITION 1.1

The *unified graph* $G_{Unified} = (M, R, E, A)$ is transformed in a directed, attributed, bipartite *kinetic graph* $G_{Kinetic} = (M_K, R_K, E_K, A_K)$ with metabolite set $M_K = M$, reaction set $R_K = R$ and edge set $E_K = E$. The attribute set in $G_{Kinetic}$ $A_K \subseteq A$ is a subset of the set in $G_{Unified}$ with A_K = {*type, stoichiometry, localization, label, concentration, rate*}, whereby the $rate$ only contains the set of rate equations J or the empty set {} (in case the *unified graph* has no assigned rate equations or has instead stochastic rates).

SUPPLEMENTARY DEFINITION 1.2

The transformation of the *unified graph* $G_{Unified} = (M, R, E, A)$ in a directed, attributed, bipartite *Petri net graph* $G_{Petri\,net} = (M_P, R_P, E_P, A_P)$ is as follows.

The metabolite set $M_P = M$, the reactions set $R_P = R \cup R_{P,new}$ equals to the set R in $G_{Unified}$ with additional reactions constructed in the process of splitting reversible reactions into an irreversible forward and an irreversible backward reaction. Each reversible reaction node (r, called original reaction) is converted into the forward reaction and an additional reaction node ($r_{P,new}$) is added for the backward reaction. The set of backward reactions ($R_{P,new}$) is defined as $R_{P,new} = \{r_{P,new} | \exists r \in R : ((m, r) \in$

$E_r \vee (r, m') \in E_r)) \wedge m, m' \in M\}$. Furthermore, the rate and the label of the original reaction with an additional sign ($'$) is assigned to the corresponding backward reaction.

The edge set in $G_{Petrinet}$ $E_P = E_{ir} \cup E_i \cup E_{P,new}$ is composed of the subsets irreversible edges E_{ir} and inhibitory edges E_i of $G_{Unified}$ and additional edges $E_{P,new}$, respectively. Due to the splitting process of reversible reactions, the forward and backward reactions will be connected with metabolites through the subsets of irreversible consumption ($E_{P,ci}$), production ($E_{P,pi}$) and inhibitory ($E_{P,i}$) edges: $E_{P,new} = E_{P,ci} \cup E_{P,pi} \cup E_{P,i}$.

Dependent on the reversible original reaction r the edge subsets are composed as follows. The subset of irreversible consumption edges is $E_{P,ci} = \{(m, r) \in E_r | m \in M \wedge r \in R\} \cup \{(m', r_{P,new}) | \exists r \in R : (r, m') \in E_r \wedge m' \in M\}$, the subset of production edges is $E_{P,pi} = \{(r, m') \in E_r | m' \in M \wedge r \in R\} \cup \{(r_{P,new}, m) | \exists r \in R : (m, r) \in E_r \wedge m \in M\}$ and the subset of inhibitory edges is $E_{P,i} = \{(m'', r_{P,new}) | \exists r \in R : (m'', r) \in E_i \wedge ((m, r) \in E_r \vee (r, m') \in E_r) \wedge m, m', m'' \in M\}$.

The type attribute ci is assigned to edges of subset $E_{P,ci}$, pi is assigned to edges of subset $E_{P,pi}$ and the type i is assigned to edges of subset $E_{P,i}$. Additionally, the stoichiometry of the original edges for the forward reaction ((r, m') and (m, r)) are assigned to respective edges for the backward reaction ($(m', r_{P,new})$ and $(r_{P,new}, m)$).

The attribute set in $G_{Petrinet}$ $A_P \subseteq A$ is a subset of the set in $G_{Unified}$ with $A_P = \{type, stoichiometry, localization, label, concentration, capacity, rate\}$, whereby the $rate$ only contains the set of stochastic rates or the empty set $\{\}$ (in case the *unified graph* has no assigned stochastic rate or has instead kinetic reaction rates).

Supplementary Figure 1 illustrates the transformation of reversible reactions of the *unified graph* in irreversible forward and backward reactions in the *Petri net graph*.

SUPPLEMENTARY DEFINITION 1.3

The *unified graph* $G_{Unified} = (M, R, E, A)$ is transformed in a directed, attributed, unipartite *metabolite graph* $G_{Metabolite} = (M_M, E_M, A_M)$ with a metabolite set $M_M = M_{cp}$ which is a subset of the set M in $G_{Unified}$. Metabolites with inhibitory interactions to reactions are not considered.

The edge set in $G_{Metabolite}$ connects metabolites with each other, which are consumed or produced in reactions of the *unified graph*. $E_M = \{(m, m') | \exists r \in R : (((m, r) \in E_r \wedge (r, m') \in E_r) \vee ((m, r) \in E_{ir} \wedge (r, m') \in E_{ir})) \wedge m, m' \in M\} \cup \{(m', m) | \exists r \in R : (m, r) \in E_r \wedge (r, m') \in E_r \wedge m, m' \in M\}$.

The transformation of, for example, reversible reactions of $G_{Unified}$ results in antiparallel edges between metabolites in $G_{Metabolite}$ which are illustrated using double-headed arrows to reduce the number of edges andto avoid edge crossings. In contrast to the *unified graph* the discrimination between substrates and products is not feasible due to reactions which reciprocal consume or produce the same metabolites.

Furthermore, if $G_{Unified}$ includes import or export reactions for metabolites which are not further connected to other reactions, the transformation in $G_{Metabolit}$ results in unconnected metabolites.

The attribute set in $G_{Metabolite}$ $A_M \subseteq A$ is a subset of the set in $G_{Unified}$ with $A_M = \{localization,\ label\}$.

Supplementary Figure 2 illustrates the transformation of irreversible, reversible, import and export reactions of the *unified graph* in the corresponding combinations of metabolites in the *metabolite graph*.

SUPPLEMENTARY DEFINITION 1.4

The *unified graph* $G_{Unified} = (M, R, E, A)$ is transformed in a directed, attributed, unipartite *reaction graph* $G_{Reaction} = (R_R, E_R, A_R)$ with a reaction set $R_R = R$.

The edge set in $G_{Reaction}$ E_R connects reactions with each other, which utilize the same metabolites in the *unified graph*. Due to the consideration of two consecutive reactions sharing the same metabolite the edge set consists of three subsets $E_R = E_{R,a} \cup E_{R,b} \cup E_{R,c}$.

The subset $E_{R,a}$ composes edges which connect a reaction r with a metabolite m with another reaction r' in the $unified\ graph$: $E_{R,a} = \{(r, r')|\exists m \in M : (r, m) \in E_{ir} \wedge type((r, m)) = pi \wedge (m, r') \in E_{ir} \wedge type((m, r')) = ci \wedge r, r' \in R\}$.

Antiparallel edges compose the subset $E_{R,b}$ which arise in the transformation of two reactions connected through the same metabolite either with two reversible edges or one reversible and one irreversible edge in the $unified\ graph$: $E_{R,b} = \{(r, r')|\exists m \in M : (((r, m) \in E_{ir} \wedge (m, r') \in E_r) \vee ((r, m) \in E_r \wedge (m, r') \in E_{ir}) \vee ((r, m) \in E_r \wedge (m, r') \in E_r)) \wedge r, r' \in R\} \cup \{(r', r)|\exists m \in M : (((r, m) \in E_{ir} \wedge (m, r') \in E_r) \vee ((r, m) \in E_r \wedge (m, r') \in E_{ir}) \vee ((r, m) \in E_r \wedge (m, r') \in E_r)) \wedge r, r' \in R\}$.

Subset $E_{R,c}$ is consists of antiparallel edges arising in the transformation of two reactions connected through the same metabolite either with two irreversible consumption edges or two irreversible production edges: $E_{R,c} = \{(r, r')|\exists m \in M : (((r, m) \in E_{ir} \wedge type((r, m)) = ci \wedge (m, r') \in E_{ir} \wedge type((m, r')) = ci) \vee ((r, m) \in E_{ir} \wedge type((r, m)) = pi \wedge (m, r') \in E_{ir} \wedge type((m, r')) = pi)) \wedge r, r' \in R\} \cup \{(r', r)|\exists m \in M : (((r, m) \in E_{ir} \wedge type((r, m)) = ci \wedge (m, r') \in E_{ir} \wedge type((m, r')) = ci) \vee ((r, m) \in E_{ir} \wedge type((r, m)) = pi \wedge (m, r') \in E_{ir} \wedge type((m, r')) = pi)) \wedge r, r' \in R\}$.

The transformation of edge subsets $E_{R,b}$ and $E_{R,c}$ results in antiparallel edges between two reactions which are illustrated with double-headed arrows to reduce the number of edges and avoid edge crossings.

Disconnected reactions in $G_{Reaction}$ arise in the transformation of reactions which utilize metabolites which are not connected with other reactions in $G_{Unified}$.

The attribute set in $G_{Reaction}$ $A_R \subseteq A$ is a subset of the set in $G_{Unified}$ with $A_R = \{label\}$.

Supplementary Figure 3 illustrates the transformation of individual and combinations of reactions of the *unified graph* in the corresponding connections of reactions in the *reaction graph*.

2 SUPPLEMENTARY FIGURES

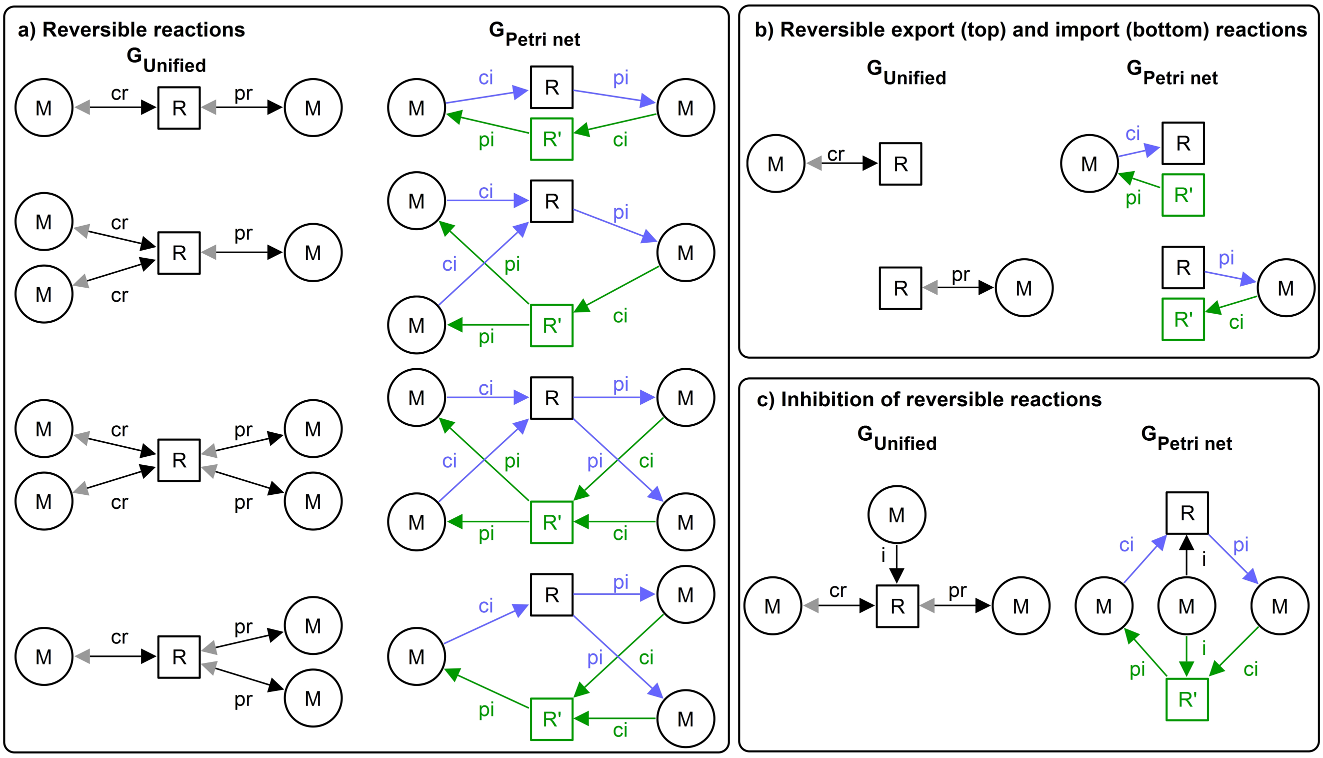

Supplementary Figure 1. Transformation of reversible reactions of the *unified graph* (left) in irreversible forward and backward reactions in the *Petri net graph* (right): a) reversible reactions, b) reversible export (top) and import (bottom) reactions, c) inhibition of reversible reactions. New edge types are assigned (blue) and additional irreversible edges (ci, pi) and reaction nodes (R', green) are added.

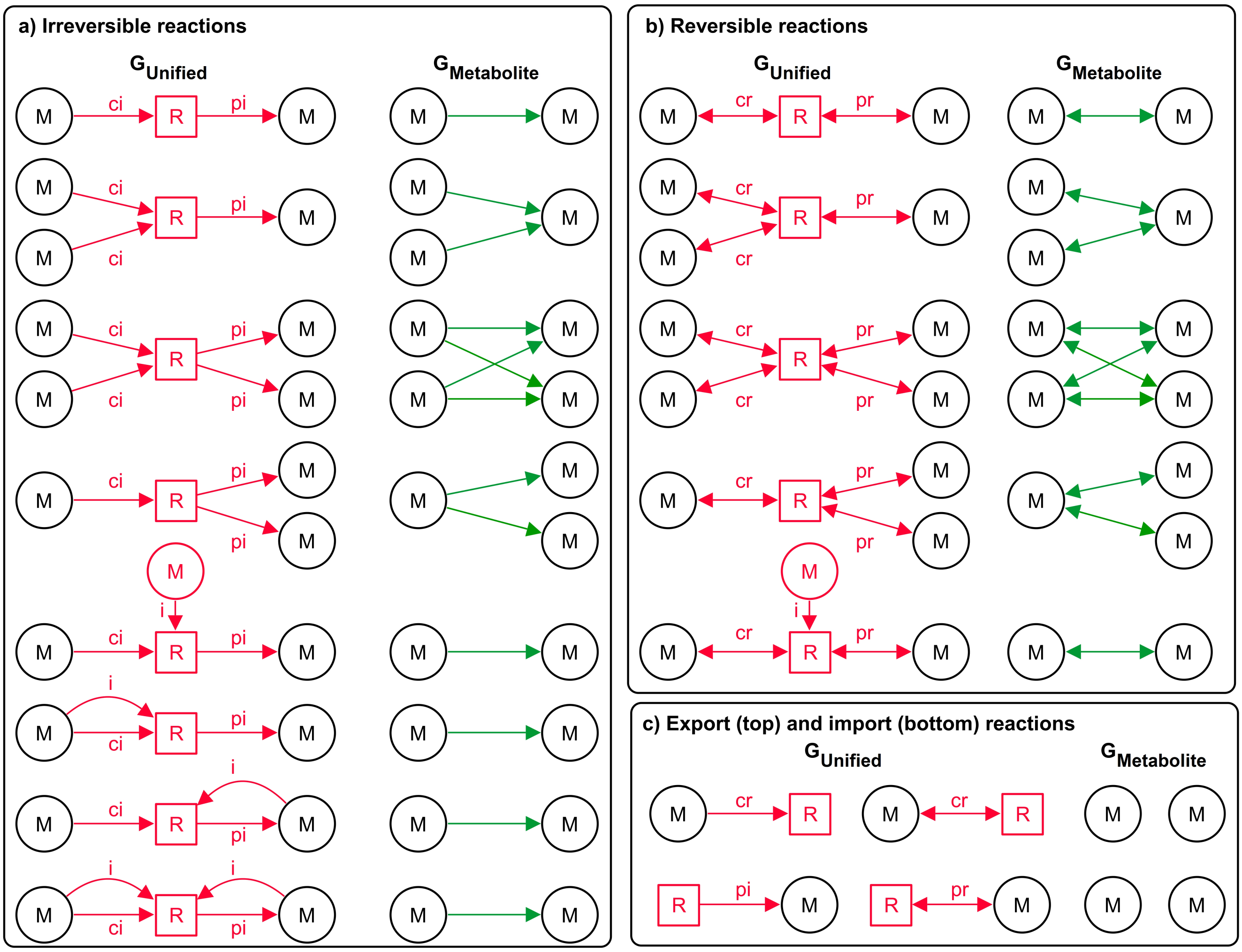

Supplementary Figure 2. Transformation of *unified graph* elements (left) in elements of the *metabolite graph* (right): a) irreversible reactions, b) reversible reactions, c) export (top) and import (bottom) reactions. Reaction nodes, edges and metabolite nodes which are connected to reactions with inhibitor edges (red) are not transformed. New directed edges between the remaining metabolite nodes are added (green). Antiparallel edges are illustrated using double-headed edges.

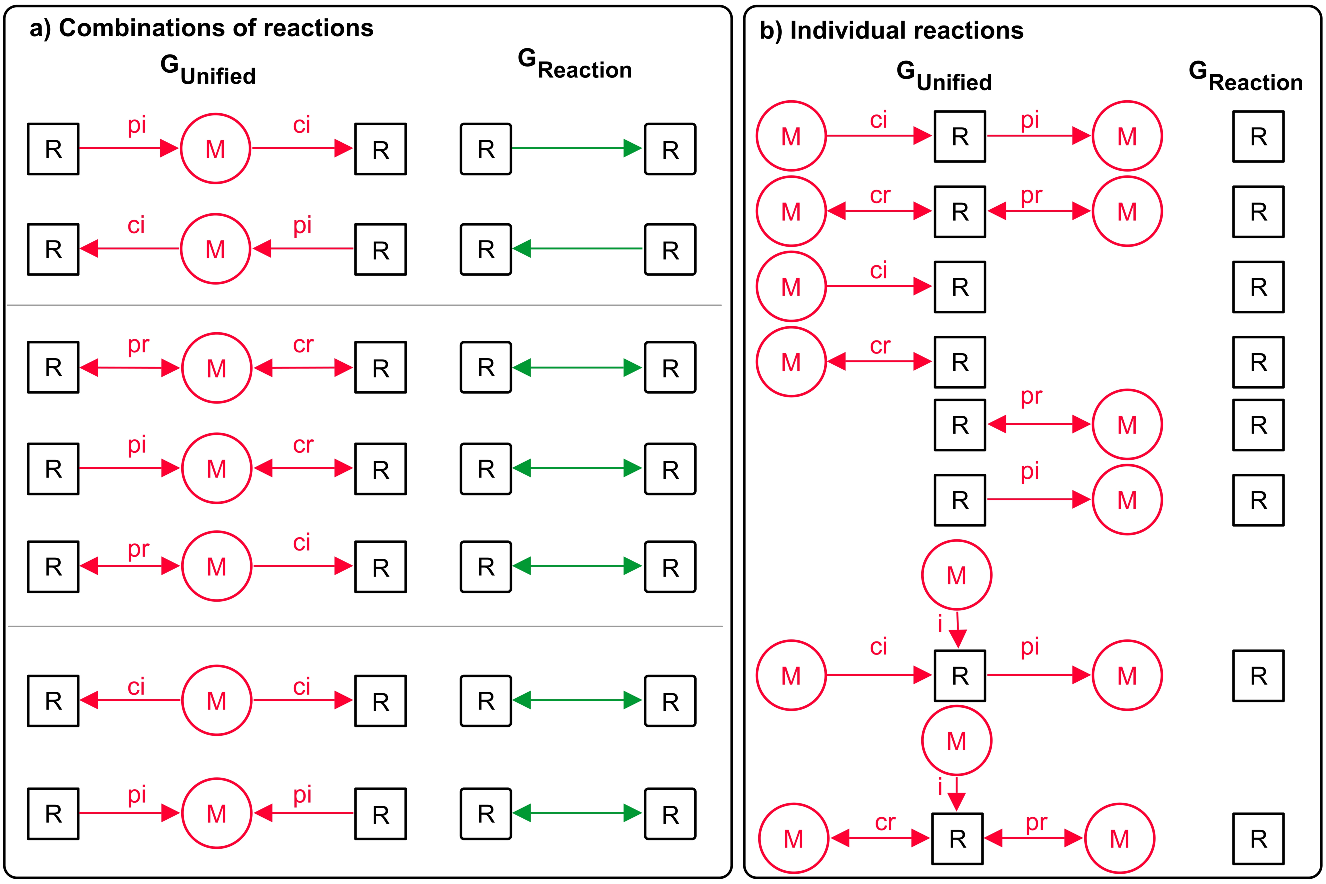

Supplementary Figure 3. Transformation of *unified graph* elements (left) in elements of the *reaction graph* (right): a) combinations of reactions, b) individual reactions. Metabolite nodes and edges (red) are not transformed. New directed edges between reaction nodes are added (green). Antiparallel edges are illustrated using double-headed edges.

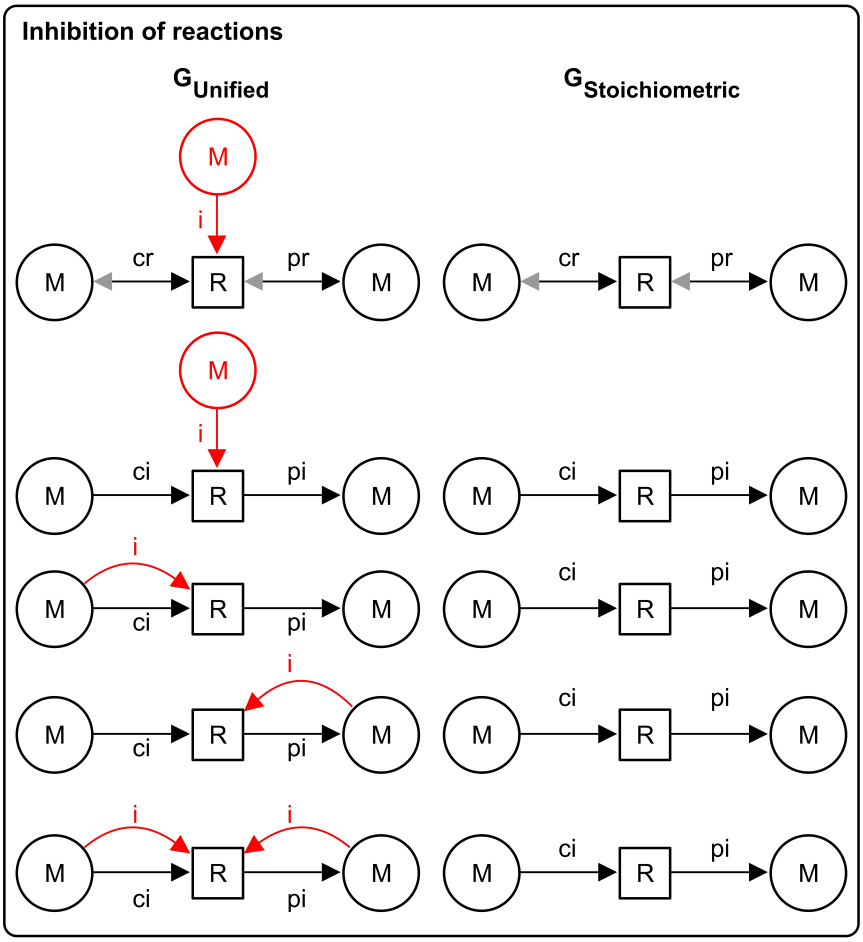

Supplementary Figure 4. Transformation of inhibited reactions of the *unified graph* (left) in reactions in the *stoichiometric graph* (right). Inhibitor edges and corresponding metabolite nodes (red) are not transformed if they are not connected to reactions through additional edges of other types than i.
